# The Synchronous Diagnosis of Multiple Myeloma (MM) and Chronic Myeloid Leukemia (CML)

**DOI:** 10.7759/cureus.73583

**Published:** 2024-11-13

**Authors:** Shinoj Pattali, Shimin Hu, Qaiser Bashir, Richard E Champlin, Muzaffar H Qazilbash

**Affiliations:** 1 Department of Hematology/Oncology, Ashtabula Regional Medical Center/Cleveland Clinic, Cleveland, USA; 2 Department of Hematopathology, University of Texas MD Anderson Cancer Center, Houston, USA; 3 Department of Stem Cell Transplantation and Cellular Therapy, University of Texas MD Anderson Cancer Center, Houston, USA

**Keywords:** autologous hematopoietic stem cell transplantation, chronic myeloid leukemia (cml), molecularly targeted therapy, synchronous multiple myeloma, treatment choices

## Abstract

The synchronous presentation of chronic myeloid leukemia (CML) and multiple myeloma (MM) is extremely rare. CML is a myeloproliferative neoplasm originating from an abnormal pluripotent hematopoietic stem cell. It is associated with the *BCR*-*ABL* fusion gene located on the Philadelphia chromosome. In contrast, multiple myeloma is a multifocal, bone marrow-based plasma cell neoplasm associated with the production of M-protein in the serum and/or urine. We present a case with a synchronous diagnosis of chronic myeloid leukemia and multiple myeloma. Both cancers were aggressively treated. The patient received autologous stem cell transplantation (ASCT) for multiple myeloma and tyrosine kinase inhibitor for chronic myeloid leukemia concurrently to achieve the complete response.

## Introduction

Chronic myeloid leukemia (CML) is a myeloproliferative neoplasm that originates from an abnormal pluripotent hematopoietic stem cell. It is associated with the *BCR*-*ABL* fusion gene located on the Philadelphia chromosome and has a worldwide annual incidence of 1-2 cases per 100,000 individuals [1]. In contrast, multiple myeloma (MM) is a multifocal, bone marrow-based plasma cell neoplasm that is associated with the production of M-protein in the serum and/or urine. The annual incidence in the United States is 1-8 per 100,000 individuals [1-3]. CML is treated with first-generation tyrosine kinase inhibitors such as imatinib in patients with low-risk scores and second-generation tyrosine kinase inhibitors such as dasatinib, nilotinib, or bosutinib in patients with intermediate- or high-risk scores. Treatment duration is usually indefinite except in a small portion of patients where treatment is discontinued after maintaining deep major molecular response (MMR) for more than two years. Multiple myeloma is treated with multiagent induction therapy, followed by melphalan chemotherapy and autologous stem cell transplantation (ASCT).

## Case presentation

**Figure 1:**
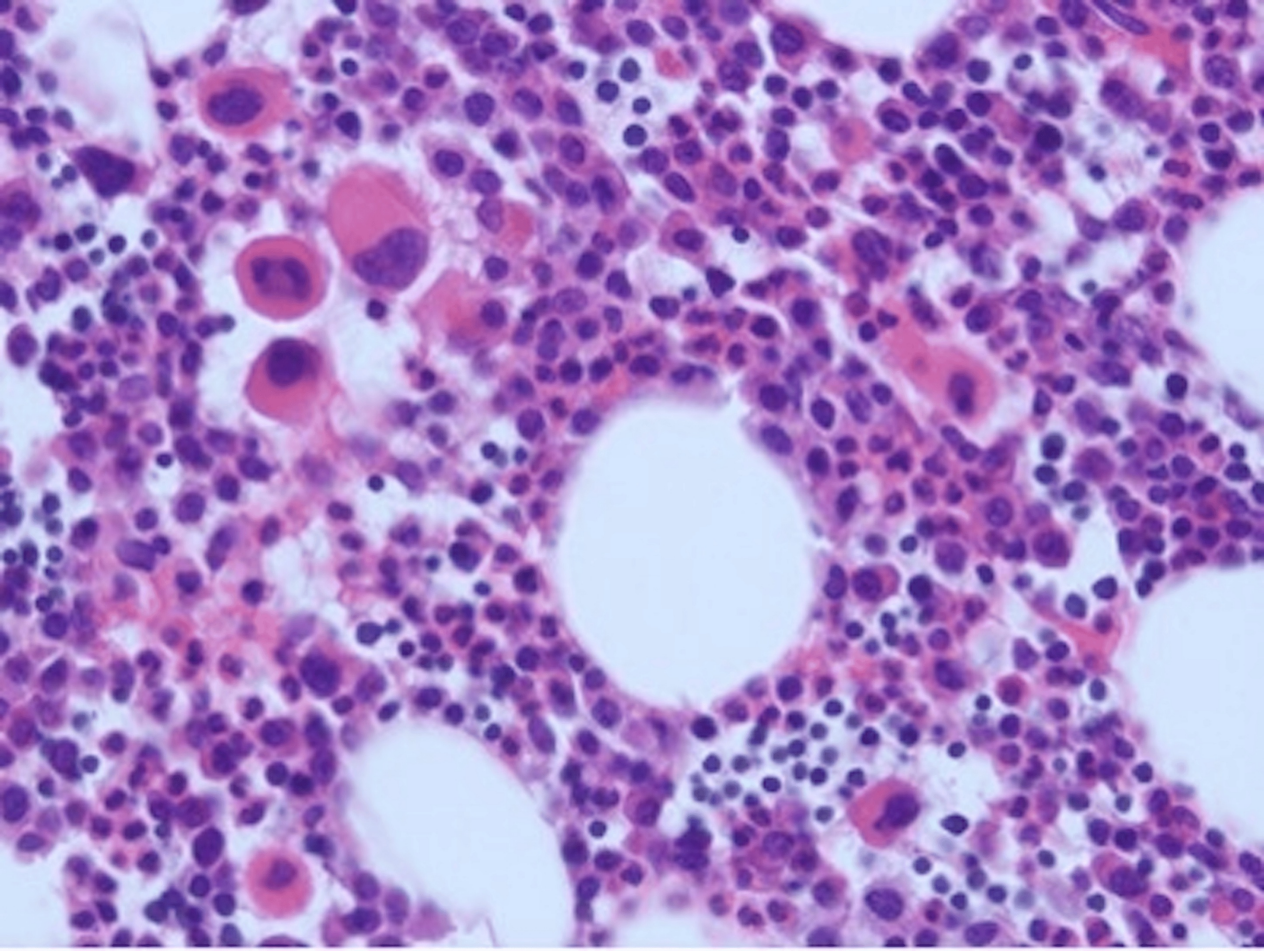
Bone marrow biopsy showing megakaryocytic hyperplasia, magnification: ×200

**Figure 2:**
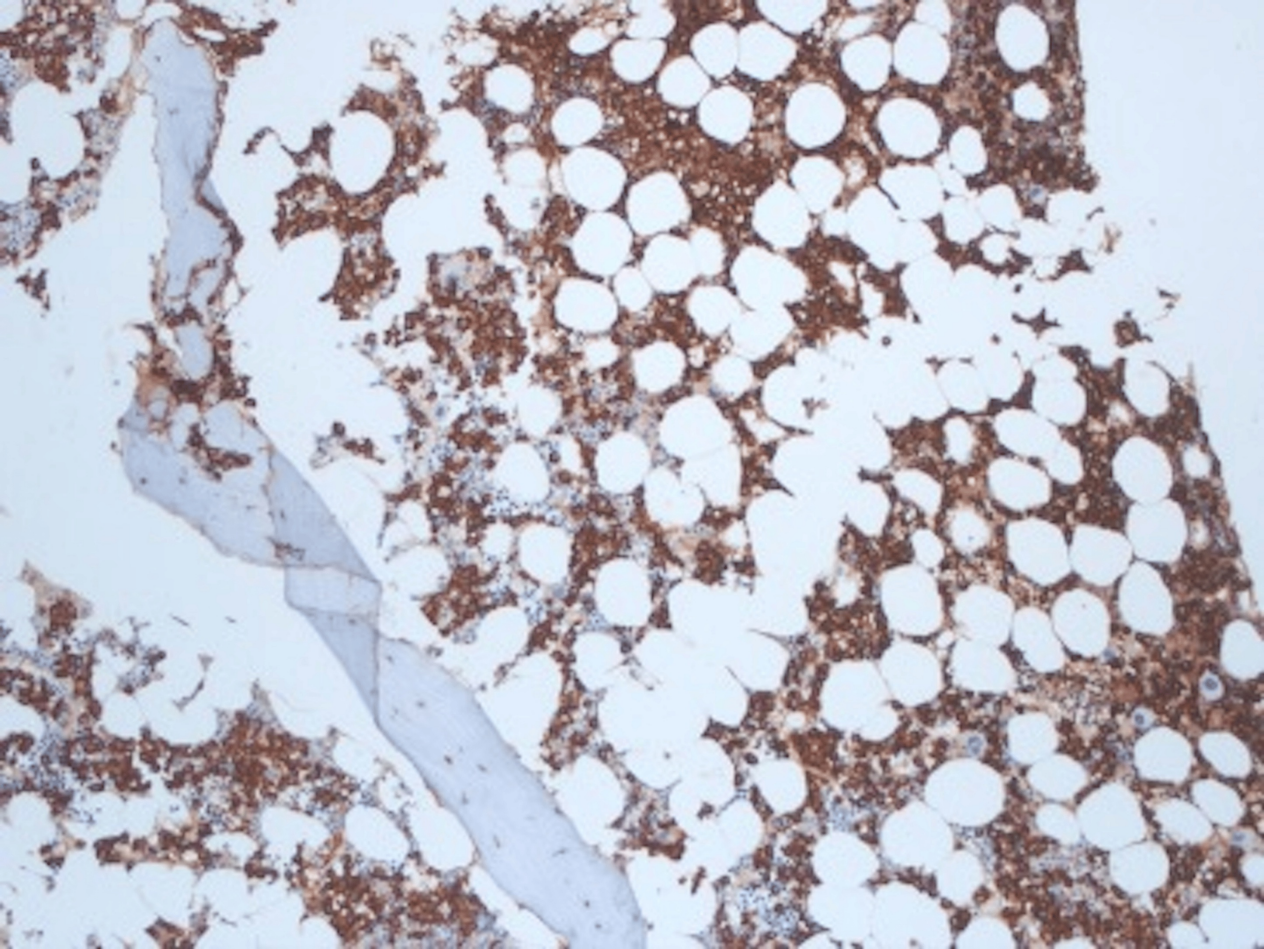
Bone marrow biopsy showing the infiltration of kappa light chain-restricted plasma cells comprising 70% of the total cellularity, magnification: ×200

**Figure 3:**
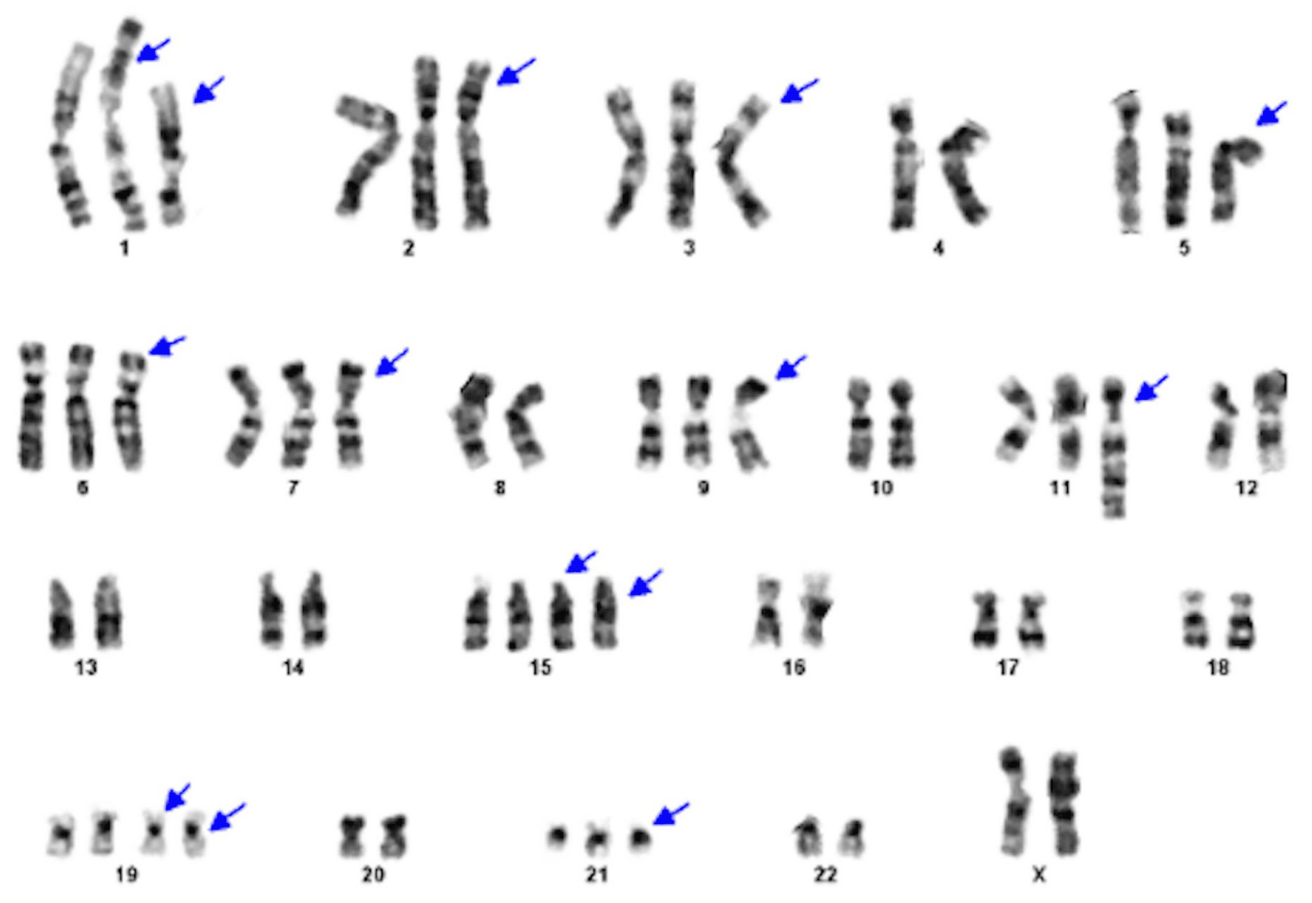
Cytogenetic studies showing hyperdiploid clone with complex karyotype and structural abnormalities involving chromosome 1 in 12/20 metaphases

**Figure 4:**
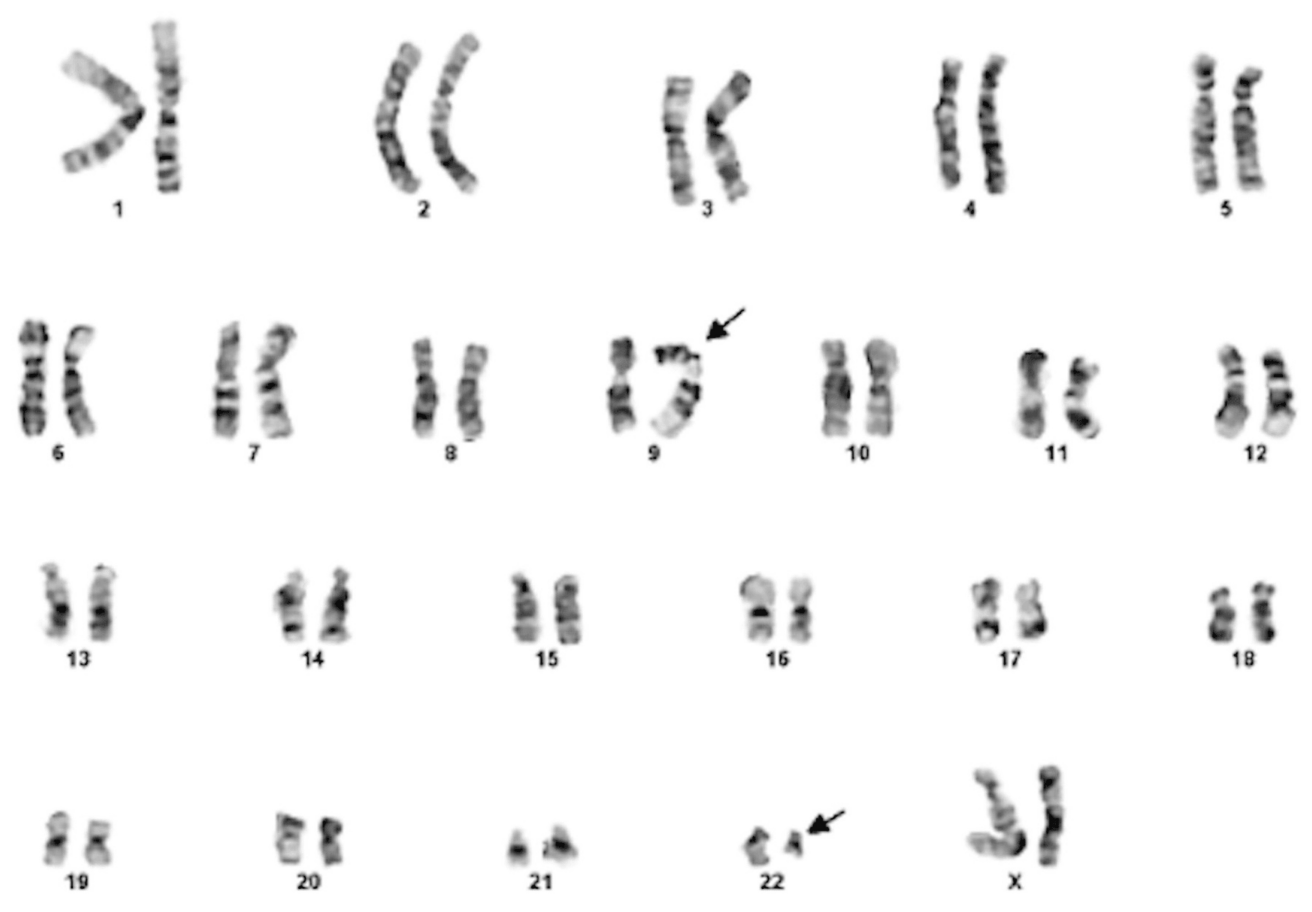
Cytogenetic study showing a clone with the Philadelphia chromosome, t(9;22)(q34;q11.2), in 5/20 metaphases

**Figure 5:**
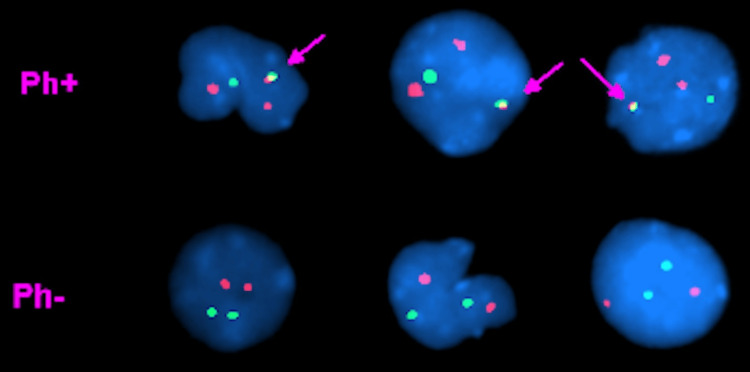
Fluorescence in situ hybridization (FISH) positive for a clone with BCR-ABL1 rearrangement Ph: Philadelphia chromosome

A 50-year-old woman presented with a three-month history of low back pain. Past medical history was significant for hypertension, pulmonary embolism, and cholecystectomy. There was no previous exposure to chemicals. The physical examination was unremarkable. Laboratory studies revealed a white blood cell count of 7.8 × 10^9^/L with 55% neutrophils and 2% immature granulocytes including metamyelocytes, myelocytes, and promyelocytes. She had mild basophilia (4%) and anemia with a hemoglobin of 10.3 g/dL. The platelet count was 269 × 10^9^/L. Her peripheral smear showed rouleaux formation. A complete chemistry panel was unremarkable with normal serum calcium and creatinine. Serum immunoglobulin G (IgG) level was elevated at 4,930 mg/dL. Serum protein electrophoresis revealed an M-protein of 4.2 g/dL, which was IgG kappa (IgG-K) on serum immunofixation. Urine protein and immunofixation electrophoresis showed kappa Bence Jones proteinuria of 606 mg in 24-hour urine volume. Beta-2 microglobulin level was 4.2 mg/L (0.7-1.8 mg/L). The bone survey showed myelomatous lesions in the skull, left scapula, L5 vertebra, and long bones. A magnetic resonance imaging (MRI) of the lumbosacral spine showed an epidural tumor involving the L5 vertebra and myelomatous lesions in the majority of the thoracic and lumbar vertebra. The patient underwent a resection of the epidural tumor. The pathology showed kappa light chain-restricted monoclonal plasma cells. The patient then underwent a bone marrow aspirate and biopsy, which showed megakaryocytic hyperplasia (Figure 1) and the infiltration of kappa light chain-restricted plasma cells comprising 70% of the total cellularity (Figure 2), confirming the diagnosis of multiple myeloma (MM). Interestingly, the cytogenetic studies showed two different clones: one hyperdiploid clone with complex karyotype and structural abnormalities involving chromosome 1 in 12/20 metaphases (Figure 3) and a clone with Philadelphia chromosome, t(9;22)(q34;q11.2), in 5/20 metaphases (Figure 4). Fluorescence in situ hybridization (FISH) was negative for immunoglobulin heavy chain (IgH) gene rearrangement, monosomy 13 or loss of *RB1* locus, and *TP53* gene deletion but was positive for a clone with *BCR*-*ABL1* rearrangement as shown in Figure 5. The quantitative real-time polymerase chain reaction (PCR) analysis of peripheral blood samples detected the presence of *BCR*-*ABL* fusion transcripts, thus establishing the diagnosis of chronic-phase chronic myeloid leukemia (CML).

The patient received induction therapy for MM with the combination of bortezomib, cyclophosphamide, and dexamethasone and achieved a very good partial response. For CML, the patient received imatinib 400 mg daily and achieved complete cytogenetic and major molecular remission with a reduction in *BCR*-*ABL*/*ABL* ratio from 15.05 to 0.07 after three months. She then underwent peripheral blood stem cell collection after mobilization with granulocyte colony-stimulating factor (G-CSF), followed by high-dose melphalan (200 mg/m^2^) and autologous hematopoietic stem cell transplantation for MM. The posttransplant course was largely uneventful. An evaluation for MM at day 100 after ASCT showed complete remission. At the last follow-up (two years since diagnosis), the patient remains on imatinib and is in complete cytogenetic and major molecular remission. She opted not to take maintenance therapy for MM but remains in complete remission.

## Discussion

The coexistence of MM and CML is an extremely rare event, with only 16 cases reported in the literature so far (Table 1) [4-19]. There is no single pattern of occurrence. In some cases, both entities were diagnosed simultaneously [6,8,10,14,18,19]; in others, CML anteceded MM [5,9,12,13,15-17]; and in a few cases, MM was diagnosed before CML [4,7,11]. In some cases where CML preceded the diagnosis of MM, the patients had been treated with imatinib, raising questions if imatinib use predisposes the patients to develop MM [13,15-17]. However, this claim has not been validated. In fact, in a recent report from MD Anderson Cancer Center, none of the 1,445 patients with CML who were treated with tyrosine kinase inhibitors, mainly imatinib, subsequently developed MM [20]. Another unanswered question in such scenarios is whether CML and MM share a common cell of origin. This hypothesis stems from the observation that CML can progress to lymphoid blast crisis with a B lineage in approximately 30% of cases [21]. However, there is little direct evidence to support this. In one report, the bone marrow cells from a patient who was simultaneously diagnosed with CML and IgG-K MM were expanded in vitro to search for a common progenitor [10]. The FISH analysis revealed that no kappa-positive plasma cells showed *BCR*-*ABL* cohybridization, while the kappa-negative cells did carry a *BCR*-*ABL* fusion signal, thus clearly distinguishing two malignant populations and favoring the coincidence of two distinct clonal disorders [10]. Our case is unique since the diagnoses were made simultaneously and aggressive treatment modalities including ASCT for MM and tyrosine kinase inhibitor for CML were given concurrently to achieve the complete response.

**Table 1:** Cases reported in literature showing the coexistence of MM and CML MM, multiple myeloma; CML, chronic myeloid leukemia; NR, not reported; 6-MP, 6-mercaptopurine; Ph, Philadelphia chromosome; IgG-K, immunoglobulin G kappa; PAM; phenylalanine mustard; XRT, radiation therapy; Mel, melphalan; Pred, prednisolone; Bu, busulfan; TG, thioguanine; Thal, thalidomide; FISH, fluorescence in situ hybridization; Dex, dexamethasone; VAD, vincristine, liposomal doxorubicin, dexamethasone; IFN-a, interferon-alpha; Cyclo, cyclophosphamide; IM, imatinib; P, prednisone; K-BJP, kappa Bence Jones proteinuria; PD, pomalidomide + dexamethasone; L-BJP, lambda Bence Jones proteinuria; IgG-L, immunoglobulin G lambda; CR, cytogenetic response; CMR, complete molecular remission; IgH, immunoglobulin heavy chain; PCR, polymerase chain reaction; IgA, immunoglobulin A; CCR, complete cytogenetic response; MMR, major molecular response; CHR, complete hematologic response; PCL, plasma cell leukemia

Reference	Diagnosis (interval between two diagnoses)	Age/sex	MM subtype	Chromosomal abnormalities	Treatment	Patient outcome at last follow-up
MacSween and Langley [4]	MM and CML (three years)	77/male	NR	Ph+	CML: 6-MP	Died three years and nine months after initial diagnosis due to leukemic transformation
Derghazarian and Whittemore [5]	CML and MM (nine years and five months)	65/female	IgG-K	Ph+ (15) and hypodiploidy (7)	CML, Bu; MM, PAM	Lost to follow-up
Boots and Pegrum [6]	CML and MM (concurrent)	58/male	IgG-K	Ph+	CML: hydroxyurea and second line, Bu + TG; MM: XRT and Mel + Pred	NR
Klenn et al. [7]	MM and CML (two years)	71/male	IgG-K	Ph+	MM, XRT and Mel + P; CML, hydroxyurea	Died three years and eight months after diagnosis
Tanaka et al. [8]	CML and MM (concurrent)	72/female	IgG-K	Ph+ (19)	CML and MM: IFN-a; second line, vindesine + Pred; third line, hydroxycarbamide + Pred	Blast crisis and death 19 months after diagnosis
Nitta et al. [9]	CML and MM (two years and nine months)	70/male	IgG-K	MM, NR; CML, Ph+	-	-
Schwarzmeier et al. [10]	CML and MM (concurrent)	66/male	IgG-K	Ph+ (50) and deletion 13q14 (FISH) only in Ph-negative kappa-restricted plasma cells	CML: hydroxyurea; second line, IFN-a; third line, Bu. MM: Mel + Pred and then Pred	Follow-up, two years: CML, in chronic phase; MM, PD
Nakagawa et al. [11]	MM and CML (three years and nine months)	47/male	K-BJP	-	-	-
Yokota et al. [12]	CML and MM (three years)	71/male	L-BJP	-	-	-
Garipidou et al. [13]	CML and MM (one year and six months)	68/male	IgG-L	Ph+ (t(9;14;22)(q34;q24;q11))	CML: IFN-a and second line, IM; MM: Mel + Pred	Follow-up, CML 26 months and MM eight months: CML, CMR; MM, CR
Wakayama et al. [14]	CML and MM (concurrent)	85/male	IgG-L	Ph+	CML and MM: Cyclo + Pred + IM	-
Galanopoulos et al. [15]	CML and MM (14 months)	76/male	IgG-K	Ph+ and hyperdiploid (FISH)	CML: IFN-a, switched to IM due to poor tolerance; MM: Mel + P	CML: blast crisis 20 months after diagnosis. Died of GIT bleeding. MM: CR
Ide et al. [16]	CML and MM (three months)	72/female	IgG-K	Ph+ (20) and plasma cells: IgH (PCR)	CML, IM; MM, none	Follow-up, nine months: CML, NR; MM, no treatment started
Michael et al. [17]	CML and MM (five years)	57/female	IgA-K	Ph+ (25) and plasma cells: no cytogenetic abnormalities	CML: IM. MM: Thal + Dex; second line, VAD; third line, bortezomib	Follow-up, six years and 11 months since initial diagnosis: CML, CCR and MMR; MM, NR
Kim et al. [18]	CML and MM (concurrent)	61/male	IgG-K	Ph+ (20)	CML, IM; MM, Dex	Follow-up, seven months: CML, CHR and partial CR. Patient died of multi-organ failure
Alvarez-Larrán et al. [19]	CML and MM (concurrent)	81/male	IgA-K	Ph+	CML and MM: Mel + P	Progression to PCL and death within a few months

## Conclusions

The synchronous diagnosis of multiple myeloma and chronic myeloid leukemia is very rare. Aggressive treatment modalities, including tyrosine kinase inhibitor for CML and multiagent induction therapy, followed by high-dose therapy and ASCT for MM, were given concurrently to achieve the complete response. To the best of our knowledge, high-dose therapy plus ASCT has never been previously reported in this scenario. While this case highlights the need to investigate the mechanism of such malignant transformation and the possibility of a common cell of origin, we are unlikely to see further studies due to the rarity of such occurrence. However, it is emphasized that aggressive therapy should not be withheld in such scenarios and long-term disease control can be expected.
